# Overactive ATP-Sensitive K^+^ Channels Compromise Lymphatic Contractile Function in Cantú Syndrome

**DOI:** 10.1093/function/zqad030

**Published:** 2023-06-08

**Authors:** Qadeer Aziz

**Affiliations:** Translational Medicine and Therapeutics, William Harvey Research Institute, Barts and the London School of Medicine and Dentistry, Queen Mary University of London, Charterhouse Square, London EC1M 6BQ, UK

## A Perspective on “Lymphatic Contractile Dysfunction in Mouse Models of Cantú Syndrome With K_ATP_ Channel Gain-of-Function”

The lymphatic system is an extensive network of vessels that runs in parallel to the blood vasculature. Both networks play crucial roles in nourishing and protecting the body. Alongside its role in “drainage,” the lymphatic system is also associated with multisystem functions and disorders, including metabolic disorders, obesity, neurological disorders, and cardiac growth and repair. Hence, a better understanding of its structural and functional physiology and pathophysiology could help in the development of future therapeutics for not only traditional lymphatic disorders such as primary and secondary lymphedema but also in the potential treatment of organ-specific functions.

Lymphatic vessels, like blood vessels, are composed of a layer of endothelial cells surrounded by a thin layer of smooth muscle cells. Electrophysiologically, lymphatic smooth muscle (LSM) exhibits intrinsic pacemaker properties that allow spontaneous action potential firing, synchronized contraction waves, and facilitation of lymph propulsion against pressure gradients. While the ion channels responsible for the pacemaker properties of LSM cells have not been clearly defined, L-type Ca^2+^ channels and ATP-sensitive K^+^ (K_ATP_) channels are present and crucial for LSM contractility and modulation of spontaneous contractility, respectively.^1^ As well as being present in tissues throughout the body, K_ATP_ channels are prominently expressed in vascular smooth muscle where they regulate vascular tone and therefore blood flow.^2–4^ Functional K_ATP_ channels are a hetero-octomeric complex of 4 pore-forming potassium channel subunits (either Kir6.1 or Kir6.2) and 4 regulatory sulphonylurea receptor subunits (either SUR1, SUR2A, or SUR2B). It is now well established that the “vascular smooth muscle” K_ATP_ channel is formed of Kir6.1 and SUR2B.^2–4^ Evidence from molecular and functional studies shows K_ATP_ channel expression and function in LSM across several species, with, as in vascular smooth muscle, Kir6.1 and SUR2B the likely subunits.^1^ Activation of K_ATP_ channels with selective K_ATP_ channel openers hyperpolarize the membrane potential of LSM cells blocking action potentials and preventing contractions. Moreover, LSM K_ATP_ channels seem to be involved in the basal function of lymphatic vessels—glibenclamide, a K_ATP_ channel blocker, depolarizes the membrane potential and increases the contraction frequency.^1^ Thus, LSM K_ATP_ channels act as negative regulators of lymphatic function where overactivity of K_ATP_ channels leads to inhibition of spontaneous contraction and reduced active lymph transport. An example of K_ATP_ channel overactivity leading to smooth muscle dysfunction is in the rare human disease, Cantú syndrome (CS).^5^ CS manifests as a result of gain-of-function (GoF) mutations in the genes encoding the “vascular smooth muscle” K_ATP_ channel, *ABCC9* (SUR2), and less commonly in *KCNJ8* (Kir6.1). CS is a multisystem disorder characterized by distinctive physical features and skeletal abnormalities, disrupted gastrointestinal motility, as well as an array of cardiovascular abnormalities.^5^ Many CS patients are also afflicted with lymphedema, some without any apparent classical symptoms of CS.^1,6^

In this issue of *Function*, Davis and colleagues use mouse models of CS to investigate the underlying mechanism of primary lymphedema with GoF mutations in the K_ATP_ channel genes *Kcnj8* and *Abcc9*.^7^ The authors used a combination of ex-vivo and in-vivo techniques to investigate whether mice expressing CS mutations in Kir6.1 and SUR2 develop lymphatic vessel dysfunction and subsequent lymphedema. The same authors have previously shown that significant lymphatic contractile dysfunction and membrane potential changes develop in smooth muscle–specific K_ATP_ GoF mice.^8^ In this study, to more faithfully mimic CS, they use mice with global K_ATP_ GoF mutations found in actual CS patients and investigate the effects of these mutations on lymphatic contractile function and the development of lymphedema. The relatively short time-scale of the experiments meant that lymphedema was difficult to detect in the CS mice even with more severe CS mutations and high gravitational load. Regardless, the evidence suggests that CS-associated GoF mutations in K_ATP_ channels lead to lymphatic contractile dysfunction with this likely resulting from an impaired active pumping mechanism. In ex-vivo preparations, CS mutations reduce the amplitude and frequency of spontaneous contractions, leading to reduced lymphatic flow. Measurements from in-vivo imaging show GoF-induced reduction in LSM excitability, leading to compromised wave entrainment and diminished active pumping. Measuring active lymphatic transport in live mice is, as the authors admit, technically challenging—the data are variable due to factors that are difficult to control, for example, inter-animal anatomical differences. However, in a more controlled ex-vivo environment, lymphatic flow in mice with CS mutations is clearly perturbed. The cumulative effect of all the above is likely to be the cause of primary lymphedema in CS patients. Moreover, the severity of lymphatic contractile dysfunction correlates well to the degree of GoF of the respective CS mutations in mice. Whether these CS mutations lead to a lymphedema phenotype that correlates with the severity of lymphatic dysfunction in CS patients remains unknown but could prove important for potential pharmacological interventions.

In summary, Davis and colleagues provide compelling evidence linking CS-associated K_ATP_ channel GoF mutations to impaired active lymphatic pumping, suggesting that this mechanism is the potential underlying cause of lymphedema in CS patients. These findings are the first example of mutations in smooth muscle ion channels leading to lymphatic contractile dysfunction and open up possibilities for a more targeted therapeutic approach for the treatment of primary lymphedema in CS patients. Proof-of-concept studies involving the use of the K_ATP_ channel blocker glibenclamide in mice and a human neonate CS subject have shown significant reversal of some CS-associated cardiovascular abnormalities.^9^ In the current study, there was some genotype-associated variability in the ability of glibenclamide to improve lymphatic function; nevertheless, the initial findings are promising and merit further investigations.^7^ Glibenclamide has off-target effects, and this requires careful consideration for potential future therapy; however, in a CS context, the relatively mild and transient hypoglycaemia observed in a human clinical study suggests a viable potential clinical therapy.^9,10^
